# EXPLORING THE RELATIONSHIP BETWEEN LONELINESS, RELIGION, AND SPIRITUALITY FOR OLDER ADULTS IN ENGLAND

**DOI:** 10.1093/geroni/igae098.0631

**Published:** 2024-12-31

**Authors:** Christina Victor

**Affiliations:** Brunel University London, London, England, United Kingdom

## Abstract

Although there is evidence linking the prevalence of loneliness with religion and/or spirituality in north America, this topic is rarely researched in the UK. This study explored the relationship between measures of religious affiliation, attendance, and practice with loneliness for older adults in England. We generated an analytic sample of 6225 adults aged 50+ who took part in wave 9 of the English Longitudinal Study of Ageing (ELSA). The exposure variables of religious affiliation, attendance and practice were measured by questions on religious identity; frequency of attendance at places of worship in the last year; and 4-point Likert scales of agreement or disagreement with religious importance, meaning and purpose, prayer and meditation, and activity. Loneliness outcome was measured using the three item University of California Los Angeles loneliness scale. The majority of our analytic sample were Christian (77%), 22% had no religion and 49% had not attended a place of worship in the last year. The prevalence of loneliness (score 6+ on UCLA scale) was 19%. Religious attendance was significantly associated with loneliness (OR =.73 CI.62-.85, p <.001), with attendance protective against feeling lonely. However, this relationship was not significant after adjustment. No relationship was observed between loneliness and religious affiliation or spirituality. Religious identity remains prevalent in older adults in England, but attendance and practice less so. Further studies in diverse populations, using validated measures of loneliness and religion, are necessary to investigate whether loneliness can be moderated or mediated by religion/spirituality alongside other determinants.

